# Protective Factors for Psychotic Symptoms Among Poly-victimized Children

**DOI:** 10.1093/schbul/sbx111

**Published:** 2017-08-31

**Authors:** Eloise Crush, Louise Arseneault, Sara R Jaffee, Andrea Danese, Helen L Fisher

**Affiliations:** 1 MRC Social, Genetic & Developmental Psychiatry Centre, Institute of Psychiatry, Psychology & Neuroscience, King’s College London, London, UK; 2 Department of Psychology, University of Pennsylvania, Philadelphia, PA; 3 Department of Child & Adolescent Psychiatry, Institute of Psychiatry, Psychology & Neuroscience, King’s College London; 4 National & Specialist CAMHS Trauma and Anxiety Disorders Clinic, South London and Maudsley NHS Foundation Trust, London, UK

**Keywords:** child abuse, childhood psychotic symptoms, home environment, IQ, resilience, social cohesion

## Abstract

**Background:**

Experiencing victimization in early life has been repeatedly shown to be associated with the emergence of psychotic symptoms in childhood. However, most victimized children do not develop psychotic symptoms and why this occurs is not fully understood. This study investigated which individual, family-level, and wider community characteristics were associated with an absence of psychotic symptoms among children at risk for psychosis by virtue of their exposure to multiple victimization experiences (poly-victimization).

**Methods:**

Participants were from the Environmental Risk Longitudinal Twin Study, a nationally representative cohort of 2232 UK-born twins. Exposure to maltreatment, bullying and domestic violence prior to age 12 was determined from interviews with mothers, children, and observations by research workers at ages 5, 7, 10, and 12. Children were interviewed about psychotic symptoms at age 12. Protective factors were measured at ages 5, 7, 10, and 12.

**Results:**

Childhood poly-victimization was associated with age-12 psychotic symptoms (OR = 4.61, 95% CI 2.82–7.52), but the majority of poly-victimized children did not report symptoms (80.7%). Having a relatively high IQ, more positive atmosphere at home, and higher levels of neighborhood social cohesion were found to be protective against childhood psychotic symptoms among poly-victimized children and also in the whole sample. However, “protected” poly-victimized children displayed elevated levels of other mental health problems compared to nonvictimized children.

**Conclusions:**

Children’s characteristics, family context, and the wider community were all found to protect children from developing early psychotic symptoms, even when they were victimized multiple times. These findings indicate targets for multilevel preventive interventions.

## Introduction

Recent literature has highlighted the presence of psychotic symptoms, such as hallucinations and delusions, in nonclinical populations,^1,2^ with around 1 in 20 children from the general population reporting them at 12 years of age.^3^ Such early experiences of psychotic symptoms have been shown to not only be distressing for children^4^ but also to increase the risk for engaging in suicidal behaviors,^5^ and for the development of schizophrenia and other psychiatric disorders in adulthood.^6,7^ It is, therefore, important to identity factors that confer protection against the manifestation of psychotic symptoms in childhood.

The vast majority of research to date has focused upon those who develop psychotic symptoms, in order to investigate associated risk factors. Adverse childhood experiences, such as physical or sexual abuse, neglect, bullying by peers, and witnessing domestic violence, appear to be a significant risk factor for the development of psychotic symptoms in childhood.^8–10^ For instance, our group previously reported on the association between different individual types of victimization, including maltreatment by an adult and bullying by peers, and the presence of psychotic symptoms in children aged 12.^8^ Exposure to more than one type of adversity in childhood (poly-victimization) has been associated with an even greater risk of developing psychotic symptoms.^8,11^ While poly-victimization has been implicated as a major risk factor, current research in this area does not address the fact that the majority of victimized children will not develop psychotic symptoms.^8^ Furthermore, it does not consider that “protective” factors could have a buffering role. Research on those who do not develop psychotic symptoms would provide valuable insights that could be harnessed to inform both the development and implementation of preventive interventions, particularly among chidren at risk for psychosis by virtue of their exposure to multiple victimization experiences (poly-victimized).^12^ Therefore, this article aims to identify individual-, family-, and community-level protective factors that are associated with a reduced likelihood of psychotic symptoms developing during childhood among poly-victimized children.

Given the lack of research exploring protective factors for psychotic symptoms, here we draw partly on the risk literature to hypothesize about factors whose absence or inverse may serve to be protective. In terms of individual-level protective factors, cognitive functioning and personality characteristics are potential candidates. It has been well-documented that children in the general population who report psychotic symptoms have a lower IQ^3,13^ and also that IQ declines in childhood have been associated with psychotic symptoms in adulthood.^14^ These findings suggest that lower IQ may be an expression of a general neurodevelopmental impairment on the pathway to psychosis.^15^ Hence, relatively high IQ levels may be protective against the development of psychotic symptoms. We have also previously found that poor executive functioning is associated with an increased risk of psychotic symptoms in children,^3^ and thus, average or higher levels of this type of cognitive functioning might be protective.

Personality characteristics, such as being shy and fearful (high harm avoidance), low cooperativeness, and a lack of ability to adapt to situations (low self-directedness), have been shown to be associated with psychotic experiences through to clinical disorder.^16–19^ It is, therefore, possible that the opposite personality traits, for example being talkative, engaging in social situations, and having natural confidence in novel situations, may be protective against the development of psychotic phenomena. Indeed, healthy adults were found to have higher persistence (eagerness and ambition) and cooperativeness (social acceptance and empathy) than patients with schizophrenia.^18^ These traits may help individuals to overcome adverse experiences, by resisting tendencies toward social withdrawal and increasing the likelihood of seeking help from others, thus protecting them from developing severe mental health problems. Although these findings were not replicated in a childhood sample.^19^ Additionally, social behaviors in childhood such as solitary play and social anxiety^20,21^ have been established as risk factors among those with a later diagnosis of schizophrenia in adulthood. It is, therefore, plausible that children who show a natural affinity for social interaction and prosocial behaviors may be less likely to develop psychotic symptoms as this may enable them to make stable friendships and develop their own supportive social networks.

Potential protective factors may also be present within the home environment in which children are brought up. A previous study conducted by our group reported that maternal warmth, sibling warmth, and a positive atmosphere at home were protective against internalizing and externalizing problems among children who had been bullied.^22^ It is possible that good relationships with family members and growing up in a nurturing and predictable environment may also be protective against psychotic symptoms among victimized children.

Community factors, outside of the home environment, such as low neighborhood social cohesion^23^ have previously been associated with psychotic symptoms emerging in both clinical and non-clinical populations, particularly in the context of victimization exposure.^24^ Therefore, living in an area where neighbors trust and get along with each other might be protective against psychotic symptoms emerging, particularly among victimized children perhaps because it increases the likelihood of others intervening if they witness maltreatment or provides more opportunities for victimized children to obtain help.

Reduced levels of social support^25^ have also previously been associated with the development of early psychotic symptoms, while having more close relationships has been suggested to protect against psychosis in adulthood.^26^ Therefore, having someone to turn for support following victimization could also be protective against the emergence of childhood psychotic symptoms.

This article utilizes prospectively collected data from a large, nationally representative cohort of UK children to explore whether individual (IQ, executive functioning, prosocial behavior, and temperament), family (atmosphere at home, maternal warmth, and sibling warmth), community (social cohesion), or cross-level (supportive adults) factors are associated with a reduced likelihood of developing psychotic symptoms among poly-victimized children. Given that poly-victimization has been associated with a range of mental health problems,^27^ we also investigated whether protected children (those exposed to poly-victimization but without childhood psychotic symptoms) were resilient to other mental health problems.

## Methods

### Study Cohort

Participants were members of the Environmental Risk (E-Risk) Longitudinal Twin Study, which tracks the development of a nationally representative birth cohort of 2232 British twin children born in England and Wales in 1994–1995. Full details about the sample are reported elsewhere^28^ and in the Supplementary Material. Briefly, the E-Risk sample was constructed in 1999–2000, when 1116 families with same-sex 5-year-old twins (93% of those eligible) participated in home-visit assessments. Families were recruited to represent the UK population of families with newborns in the 1990s, based on residential location throughout England and Wales and mothers’ age. Teenaged mothers with twins were over-selected to replace high-risk families who were selectively lost to the register through nonresponse. Older mothers having twins via assisted reproduction were underselected to avoid an excess of well-educated older mothers. E-Risk families are representative of UK households across the spectrum of neighborhood-level deprivation (see online Supplementary Material). The sample comprised 56% monozygotic and 44% dizygotic twin pairs, and sex was evenly distributed within zygosity (49% male). Follow-up home-visits were conducted when children were aged 7, 10, and 12 (participation rates were 98%, 96%, and 96%, respectively). The Joint South London and Maudsley and the Institute of Psychiatry Research Ethics Committee approved each phase of the study. Parents gave informed consent and children gave assent.

### Measures

#### Childhood Psychotic Symptoms

E-Risk families were visited by mental health trainees or professionals when children were aged 12.^3^ Each child was privately interviewed about seven psychotic symptoms pertaining to delusions and hallucinations. Items and interviewer notes were assessed by a psychiatrist expert in schizophrenia, a psychologist expert in interviewing children, and a child and adolescent psychiatrist to verify the validity of the symptoms. This interview and coding procedure has been described in detail previously^3^ and in Supplementary Material. At age 12, the majority of children in the sample had complete data on psychotic symptoms (*N* = 2127/2146, 99.1%). A total of 5.9% of children reported experiencing at least one definite psychotic symptom (*N* = 125). This is similar to the prevalence of psychotic symptoms in other community samples of children and adolescents.^2,15,29,30^

#### Other Mental Health Problems

At age 12, children completed the 10-item version of the Multidimensional Anxiety Scale for Children.^31^ Those who scored at or above the 95th centile (raw score of 13 or more) constituted the “extreme” anxiety group. We used scores of 20 or more on the Children’s Depression Inventory^32^ completed by children at age 12, to indicate clinically-significant depressive symptoms.^33^ We derived diagnoses of conduct disorder at age 12 on the basis of mothers’ and teachers’ reports of children’s behavior problems using the Achenbach family of instruments and additional *Diagnostic and Statistical Manual of Mental Disorders*, Fourth Edition, items assessing conduct disorder which have previously been described.^34^

#### Childhood Poly-victimization

Exposure to several types of victimization was assessed repeatedly when the children were 5, 7, 10, and 12 years of age and dossiers have been compiled for each child with cumulative information about exposure to domestic violence between the mother and her partner, frequent bullying by peers, physical maltreatment by an adult, sexual abuse, emotional abuse and neglect, and physical neglect. Each form of victimization was rated by coders as “0” not present; “1” probable harm, occasionally present, or evidence of only minor incidents; or “2” definite harm, frequently present, or evidence of severe incidents. Poly-victimization was defined as experiencing two or more types of victimization that were coded as “2” before age 12 (*N* = 140, 6.6%) compared to only one type or none (*N* = 1986, 93.4%). We utilized a conservative cut-off of “2” in order to increase the likelihood that we were capturing “true” incidences of victimization (rather than occasional teasing or minor forms of punishment such as being smacked on the bottom), because more severe incidences of victimization have been suggested to be more likely to be recalled accurately.^35^ Moreover, severe victimization has been associated with the highest risk of later mental health problems.^36^ Details about these measurements have been reported previously^37,38^ and are provided in Supplementary Material.

#### Individual-Level Protective Factors

The Wechsler Preschool and Primary Scale of Intelligence Revised (WPPSI)^39^ was used to assess IQ at age 5. Children were administered two subtests (Vocabulary and Block Design), and IQ scores were prorated following procedures described previously^40^ and then standardized with a mean of 100 and standard deviation of 15.

Executive function was measured at age 5 as the mean score of three separate tasks: Mazes,^41^ a WPPSI subtest; Day-Night,^42^ a nonverbal analog of the Stroop task; and Sentence Working Memory, based on the Baddeley model of working memory;^43,44^ after converting each scale to a common metric. The resulting combined score was standardized with a mean of 100 and standard deviation of 15.

After the age-5 home visits, research workers rated each twin on 25 different behavioral characteristics that assessed children’s style of approach and response to the testing session. The behavioral characteristics were derived from scales initially used to rate children enrolled in the American Collaborative Study on Cerebral Palsy, Mental Retardation, and Other Neurological Disorders of Infancy and Childhood^45^ and were modified for use in the Dunedin Health and Development Study.^46^,^47^ The current study used the measure for “Approach” as it captures contrasting traits to those associated with the broader psychosis phenotype.^19^ This temperament measure was made up of six items including quick adjustment, friendliness, self-confidence, talkativeness, easy separation, and smiling and laughter (internal consistency: α = 0.90).

Prosocial behavior was derived using 10 items from the Revised Rutter Parent Scale for School-Age Children^48^ to extract a prosocial score where the items were summed^49^ for children at age 5 (internal consistency: α = 0.77). Items included “considerate of other people’s feelings,” “kind to younger children,” and “shares out treats with friends.” Questionnaires were completed by both mothers and teachers; the total scores were combined and then averaged to provide a single score.

#### Family-Level Protective Factors

Maternal warmth was assessed using procedures adapted from the Five-Minute Speech Sample method.^50^ Mothers were asked to speak for 5 min about each of their children when they were aged 5 and again at age 10. Warmth was coded on a six-point scale from no warmth (complete absence of warmth) to high warmth (definite warmth, enthusiasm, interest in, and enjoyment of the child). Two trained raters, blind to all other E-Risk Study data, coded the tapes of the mothers’ speech sample (inter-rater agreement: *r* = 0.90). The maternal warmth scores at ages 5 and 10 were combined, as they were significantly correlated (*r* = 0.37, *P* < .001), and then averaged to provide a single score.

Mothers were asked a series of questions about the quality of their children’s relationship with one another when the children were aged 7 and 10.^51^ Mothers responded on a three-point scale to six questions (eg, “do your twins love each other,” “do both your twins do nice things for each other”). The internal consistency reliability score at age 7 was 0.77 and at age 10 was 0.80. The sibling warmth scores at ages 7 and 10 were combined, because they were significantly correlated (*r* = 0.57, *P* < .001), and then averaged to provide a single score.

The creation of the atmosphere at home measure has been previously documented.^52^ It was derived from the Coder’s Impression Inventory, which is based on the Home Observation for Measurement of the Environment^53^ and the University of Washington Parenting Clinic Questionnaire (Parent–Child Observations).^54^ The Coder’s Impression Inventory was rated immediately following the study visit at ages 7 and 10 by interviewers who had undergone 4-day training. This measure comprised items representing the state of the home (eg, “Are visible rooms of the house clean?”), stimulation (eg, “Is the children’s art displayed in the home?”), happiness (eg, “Is this a happy home?”) and chaos (eg, “Is the house chaotic or overly noisy?”). The internal consistency at age 7 was α = 0.77 and α = 0.79 at age 10. The average of the overall atmosphere at home scores at ages 7 and 10 was used for analysis because they were significantly correlated (*r* = 0.64, *P* < .001). The four subscales were also examined separately using an average of the scores at 7 and 10.

#### Community-Level Protective Factors

We assessed social cohesion^55^ when children were aged 5 by asking mothers five questions, including whether their neighborhood was closeknit, whether neighbors shared values, and whether neighbors trusted and got along with each other. We derived a total score by summing the answers to all five questions (internal consistency: α = 0.83), with higher scores indicative of greater social cohesion.

#### Cross-Level Protective Factors

The presence of a supportive adult was assessed at age 12 when children were asked questions about whether they had a stable adult figure to rely on for basic needs and support (eg, “there is an adult who I can tell almost anything to,” “there is an adult who I can go to if I am in trouble”). Participants answered not true (0), sometimes true (1), or true (2). We derived a total score by summing responses to 13 items (internal consistency: α = 0.85). The questions did not ask the child to specify who the adult was, and thus, this could have been someone within or outside of their family.

#### Family-Level Confounders

Family socioeconomic status (SES) was measured via a composite of parental income (total household), education (highest for mother/father), and occupation (highest for mother/father) when children were aged 5^56^ and was categorized into tertiles (ie, low-, medium-, and high-SES). Family psychiatric history was assessed when children were aged 12. In private interviews, mothers reported on family history of DSM disorders,^57^ which was converted to a proportion (0–1.0) of family members with a history of psychiatric disorder.

### Statistical Analysis

Analyses were conducted in STATA 11.2 (Stata-Corp, College Station, TX). Because each study family contains two children, all statistical analyses were corrected conservatively for the nonindependence of twin observations by using tests based on the Huber/White variance estimator.^58^ Application of this technique allows for the relaxation of the assumption of independence of observations by penalizing estimated standard errors and therefore accounting for the dependence in the data due to analyzing sets of twins. We used binary logistic regression to test the associations between (i) childhood poly-victimization and age-12 psychotic symptoms in the whole sample; and (ii) individual-, family-, and community-level protective factors and age-12 psychotic symptoms in the poly-victimized group. We also tested for interactions between significant protective factors and poly-victimization in the whole sample using logistic regression to examine whether these factors were specifically protective in relation to poly-victimization exposure. All of these analyses were adjusted for gender, family SES and family psychiatric history. Additionally, we examined whether the poly-victimized children who did not develop psychotic symptoms were more likely to have anxiety, depression, or conduct disorder at age 12, using binary logistic regression and controlling for gender and family SES.

## Results

### Is Poly-victimization in Childhood Associated With Age-12 Psychotic Symptoms?

Psychotic symptoms at age 12 were more commonly reported by children who were exposed to multiple types of victimization than in those who were not poly-victimized (19.3% vs 4.9%, respectively; OR = 4.61, 95% CI 2.82–7.52, *P* < .001). This association remained after controlling for family SES (OR = 4.22, 95% CI 2.50–7.10, *P* < .001) and family history of mental health problems (OR = 3.72, 95% CI 2.20–6.29, *P* < .001) and did not significantly differ for boys and girls (interaction: OR = 1.72, 95% CI 0.63–4.67, *P* = 0.286), and therefore, all further results will be presented for both sexes together.

Among poly-victimized children (*N* = 140), those who did and did not develop psychotic symptoms were comparable in terms of the total number of victimization experiences they encountered (χ^2^(3) = 5.807, *P* = .121). The two groups were also statistically comparable in terms of the types of victimization they experienced (emotional abuse and neglect: psychotic symptoms absent 41% vs present 44%, χ^2^(2) = 0.141, *P* = .932; physical abuse: 60% vs 56%, χ^2^(2) = 0.355, *P* = .837; physical neglect: 27% vs 26%, χ^2^(2) = 1.567, *P* = .457; sexual abuse: 4% vs 15%, χ^2^(2) = 4.058, *P* = .131; bullying: 45% vs 59%, χ^2^(2) = 2.703, *P* = .259; domestic violence: 78% vs 59%, χ^2^(2) = 4.748, *P* = .093).

### Are Individual, Family, and Community-Level Factors Associated With the Absence of Age-12 Psychotic Symptoms Among Poly-victimized Children?

We first explored whether the potentially protective factors were operating in the context of exposure to poly-victimization. A relatively high IQ and more positive atmosphere at home were found to be associated with a reduced likelihood of psychotic symptoms emerging among children exposed to poly-victimization (indicated by OR < 1; table 1). Higher levels of neighborhood social cohesion showed a protective trend but fell short of statistical significance (*P* = .090). The associations were almost identical and remained statistically significant when controlling for each other (IQ: OR = 0.96, 95% CI 0.93–1.00, *P* = .043; atmosphere at home: OR = 0.93, 95% CI 0.87–1.00, *P* = .041), indicating that their effects were independently protective against childhood psychotic symptoms in the context of poly-victimization. In terms of the atmosphere at home subscales, only the physical state of the home (OR = 0.83, 95% CI 0.70–1.00, *P* = .044) was found to be independently protective against psychotic symptoms, after controlling for IQ. The subscales relating to the stimulating nature (OR = 0.89, 95% CI 0.73–1.07, *P* = .208), happiness (OR = 0.78, 95% CI 0.59–1.02, *P* = .070), and predictability and calmness (OR = 0.80, 95% CI 0.60–1.06, *P* = .120) of the home environment were not found to be independently protective. None of the other individual-, family-, or community-level factors appeared to be significantly protective in this subsample (table 1).

**Table 1.** Associations Between Potential Protective Factors and Age-12 Psychotic Symptoms Among Children Exposed to Poly-victimization

	Poly-victimized Children (*N* = 140)			
Childhood Factors	Psychotic Symptoms Absent *n* = 113 M (SD)	Psychotic Symptoms Present *n* = 27 M (SD)	Unadjusted (95% CI)	Adjusted OR^a^ (95% CI)
IQ	93.0 (13.3)	86.4 (12.2)	**0.96 (0.93–0.99**)	**0.96 (0.93–0.99**)
Executive function	96.8 (16.2)	92.6 (15.5)	0.98 (0.96–1.01)	0.98 (0.96–1.01)
Temperament (approach)	9.0 (3.4)	8.6 (3.6)	0.97 (0.86–1.10)	0.95 (0.82–1.07)
Prosocial behavior	26.1 (6.6)	23.9 (6.5)	0.95 (0.89–1.02)	0.94 (0.88–1.02)
Maternal warmth	2.9 (0.9)	2.9 (1.1)	0.97 (0.59–1.59)	0.92 (0.54–1.53)
Sibling warmth	8.9 (2.1)	9.4 (1.8)	1.15 (0.92–1.45)	1.15 (0.91–1.44)
Atmosphere at home	18.6 (7.3)	15.5 (6.3)	**0.94 (0.89–0.99**)	**0.93 (0.87–0.99**)
Supportive adult	22.7 (4.5)	21.3 (5.7)	0.94 (0.87–1.03)	0.94 (0.86–1.02)
Social cohesion	5.8 (3.3)	4.5 (3.4)	0.89 (0.77–1.01)	0.88 (0.76–1.02)

CI, confidence interval. IQ, intelligence quotient. M, mean. OR, odds ratio. SD, standard deviation.

^a^Adjusted for family socioeconomic status, family psychiatric history, and child’s gender. All analyses account for the nonindependence of twin observations.

Bold text indicates *P* < .05.

### Are Poly-victimized Children Who Do Not Develop Psychotic Symptoms Also Protected Against Other Mental Health Problems at Age 12?

In the group of children who did not develop age-12 psychotic symptoms (*N* = 2002), poly-victimized children were more likely than those who were not poly-victimized to have conduct disorder (24.8% vs 4.1%, respectively; OR = 3.94, 95% CI 2.02–7.67, *P* < .001), clinically-relevant depression (10.6% vs 2.3%; OR = 3.79, 95% CI 1.71–8.36, *P* = .001), and extreme levels of anxiety (11.5% vs 5.0%; OR = 2.40, 95% CI 1.19–4.86, *P* = .015) at age 12. Thus, indicating that poly-victimized children who were protected against psychotic symptoms were not resilient more broadly to other mental health problems.

### Are These Protective Factors Specific to Poly-victimized Children?

We further tested for interaction effects to understand whether the factors identified were particularly protective in relation to poly-victimization exposure. We did not find any of these interactions to be significant: IQ (interaction OR = 0.99, 95% CI 0.95–1.02, *P* = .520), positive atmosphere at home (interaction OR = 0.99, 95% CI 0.93–1.06, *P* = .847), or social cohesion (interaction OR = 0.98, 95% CI 0.84–1.14, *P* = .786). Indeed, having a relatively high IQ, more positive atmosphere at home, and also higher levels of neighborhood social cohesion were also associated with a reduced likelihood of psychotic symptoms in the whole sample (table 2). All three of these associations held after controlling for the other significant factors, suggesting that higher IQ (OR = 0.98, 95% CI 0.96–0.99, *P =* .001), a more positive atmosphere at home (OR = 0.95, 95% CI 0.92–0.98, *P =* .003), and increased social cohesion (OR = 0.92, 95% CI 0.86–0.98, *P* = .012) were all independently associated with a reduced likelihood of childhood psychotic symptoms in the whole sample. In terms of the atmosphere at home subscales, the physical state (OR = 0.83, 95% CI 0.75–0.91, *P* < .001), stimulating nature (OR = 0.91, 95% CI 0.83–0.99, *P* = .028), and predictability and calmness (OR = 0.75, 95% CI 0.65–0.86, *P* < .001) of the home environment were all found to be independently associated with a reduced likelihood of psychotic symptoms, after controlling for IQ and social cohesion. The subscale relating to happiness within the home (OR = 0.87, 95% CI 0.74–1.03, *P* = .114) was not found to be independently associated.

**Table 2.** Associations Between Potential Protective Factors in Childhood and Age-12 Psychotic Symptoms in the Full Sample

	Whole Sample (*N* = 2127)			
Childhood Factors	No Psychotic Symptoms *n* = 2002 M (SD)	Psychotic Symptoms *n* = 125 M (SD)	Unadjusted (95% CI)	Adjusted OR^a^ (95% CI)
IQ	100.5 (14.9)	93.0 (14.6)	**0.97 (0.95–0.98**)	**0.97 (0.96–0.98**)
Atmosphere at home	26.0 (5.4)	22.7 (6.6)	**0.92 (0.90–0.94**)	**0.93 (0.90–0.96**)
Social cohesion	7.7 (2.7)	6.5 (3.2)	**0.87 (0.82–0.93**)	**0.89 (0.84–0.96**)

^a^Adjusted for family socioeconomic status, family psychiatric history, and child’s gender. All analyses account for the nonindependence of twin observations.

Bold text indicates *P* < .05.

## Discussion

To our knowledge, this is the first study to investigate individual-, family-, and community-level factors that may protect children from developing psychotic symptoms. Having a relatively high IQ and more positive atmosphere at home were associated with a reduced likelihood of reporting psychotic symptoms at age 12, even when children had been victimized in multiple ways. We also found strong protective trends for children who lived in areas with higher levels of neighborhood social cohesion in the poly-victimized group.

First, in terms of individual-level protective factors, our findings suggest that a relatively high IQ was associated with a reduced likelihood of developing psychotic symptoms, both in the high-risk group exposed to polyvictimization and in the whole sample. This may indicate that such children do not manifest early neurodevelopmental impairments that have previously been linked to development of schizophrenia in adulthood.^21^ In terms of potential mechanisms, it is possible that a relatively high IQ could facilitate the development of effective coping styles that have previously been found to bolster resiliency against mental health problems,^59,60^ and therefore, might also be protective against the onset of psychotic symptoms. Higher IQ may also promote cognitive flexibility that has been associated with an absence of psychopathology.^61^

A more positive atmosphere at home was also found to be protective in the poly-victimized group and among the general population, which is consistent with prior research that has highlighted the protective effects of family stability in the context of adversity,^62^ and how more chaotic living situations can increase the risk of early psychotic symptoms^63^ and adult psychosis.^64^ Given that some types of victimization that children are exposed to may take place outside of the home, the home environment may provide children with a safe, nurturing environment that acts as a refuge, which, in turn, may lessen the harmful effects of their experiences on cognitive and emotional processes.^65^ Even for children where victimization does take place within the home, if there are other positive aspects to the environment, then children may be able to benefit from these, perhaps by buffering their overall stress response.^66^ Our atmosphere at home measure captured both physical (eg, noise, cleanliness and child-focused stimulation) and emotional (ie, whether the home felt like a happy environment) aspects of the home environment and secondary analyses suggested that the physical attributes were more protective. It would be useful for future studies to investigate further which specific elements are protective in order to inform prevention strategies.

In terms of community factors, higher levels of neighborhood social cohesion were shown to have a protective trend in relation to childhood psychotic symptoms in the poly-victimized group and also independently among the general population. This is in keeping with previous studies that have found supportive relationships between neighbors promote positive parenting practices and may protect against the adverse effects of maltreatment.^67,68^ Moreover, general perceptions of a supportive environment may facilitate children to more quickly obtain help with any distress they are experiencing and cope with it better,^69^ as well as potentially accessing normalizing explanations for their anomalous experiences that may reduce the likelihood of developing clinically-relevant psychotic symptoms.^70^

Our finding that having a higher IQ and more positive atmosphere at home (and to a nonsignificant degree, higher social cohesion) were protective in the context of poly-victimization is important because such children are at much higher odds of developing psychotic symptoms.^8,11^ Furthermore, this poly-victimized subgroup represent a much smaller number of individuals which is more practical in terms of targeting interventions. Assuming that our results are replicated in other cohorts, our findings could be utilized to inform which individuals should be targeted with preventive interventions, as well indicating the content or focus of such interventions, eg, engaging with families and educating parents on the importance of a structured positive home environment.

Notably, we also found that poly-victimized children in this sample who did not develop psychotic symptoms could not be considered to be broadly “resilient” to other mental health problems because they had higher rates of conduct disorder, depression, and anxiety symptoms compared to their peers who were not exposed to multiple types of victimization. Given that poly-victimization is associated with a range of mental health problems,^27^ it is not surprising that the poly-victimized group showed elevated levels of other types of psychopathology. Our findings suggest that there may be different protective factors operating in relation to different mental health problems. A prior study^22^ in our cohort found sibling and maternal warmth to be protective in relation to emotional and behavioral problems at age 12 among children exposed to bullying victimization, whereas the current study did not find either factor to be protective against psychotic symptoms in the context of poly-victimization. Further research is required to establish which factors protect vulnerable children against a wider range of mental health problems.

All factors found to be protective in our poly- victimized group were also found to be associated with a reduced likelihood of age-12 psychotic symptoms in the whole sample. While it is interesting that factors continued to be protective among children at high risk, these factors were not unique or disproportionately protective in the context of poly-victimization, as demonstrated by a lack of significant interaction effects. In the absence of any other studies in this area, we would welcome replication of our findings in order to establish whether other cohorts find similar results.

## Limitations

Some limitations warrant consideration. First, despite this being a reasonably large cohort, the numbers of poly-victimized children was fairly small and this may have limited our ability to detect some associations between the proposed protective factors and a reduced likelihood of developing psychotic symptoms. These analyses thus warrant replication in even larger population-based cohorts. Second, we only focused on childhood psychotic symptoms and therefore cannot be certain whether children unaffected at this age develop psychotic symptoms later. Thirdly, while this study was able to identify specific individual-, family-, and community-level factors that were associated with a reduced likelihood of childhood psychotic symptoms, we were not able to investigate whether specific levels or ranges of these factors were associated with the lowest likelihood of psychotic symptoms emerging given the size of the poly-victimized group. However, this study does provide a useful starting point for future research to consider the relationships between different levels of each protective factor and the absence of psychotic symptoms among poly-victimized children. Fourth, childhood psychotic symptoms are associated not only with later development of schizophrenia but also other mental health problems,^6,7^ and thus, the findings cannot specifically be generalized to clinically-relevant psychosis in adults. Fifth, we were not able to account for the specific timings of victimization exposure^71^ nor was information available regarding attachment style,^72,73^ and thus, we were unable to explore the potential role of these factors in our analyses. We also used a conservative cutoff to indicate the presence of victimization, which may have resulted in an underestimation of the true poly-victimization rates. Finally, the E-Risk cohort comprises twins, and whether findings from twin studies generalize to singletons is sometimes contested. However, the children in our study are representative of singletons for the prevalence of psychotic symptoms^2,15,28,29^ and representative of UK families in terms of geographic and socioeconomic distribution.^74^

## Conclusion

A relatively higher IQ, a more positive atmosphere at home, and higher neighborhood social cohesion were found to be associated with an absence of psychotic symptoms at age 12 in this general population sample, even among those exposed to multiple forms of victimization. In terms of practical implications, these findings suggest we should aim to target prevention efforts toward the smaller “higher risk” group of poly-victimized children given that resources are often severely limited. If these findings are replicated in other large population-based cohorts, then it would be useful for clinicians, educators, and community workers to develop and test interventions that could improve children’s home and community environments and support their cognitive development to hopefully increase their resiliency to childhood psychotic symptoms.

## Supplementary Material

Supplementary material is available at *Schizophrenia Bulletin* online.

## Funding

The E-Risk Study is funded by the UK Medical Research Council (G1002190). Additional support was provided by the National Institute of Child Health and Human Development (HD077482); the Jacobs Foundation; the British Academy (SQ140024 to Helen L. Fisher); a Medical Research Council Studentship to Eloise Crush; and an MQ Fellows Award to Helen L. Fisher (MQ14F40). Louise Arseneault is the Mental Health Leadership Fellow for the UK Economic and Social Research Council.

## Supplementary Material

Supplementary_Materials
