# Supplementary material for: Protective Factors for Psychotic Symptoms Among Poly-victimized Children
Source: Schizophr Bull. 2017 Aug 31;44(3):691–700. doi: 10.1093/schbul/sbx111 (PMC5890453; doi:10.1093/schbul/sbx111)
Supplement: Supplementary_Materials [file sbx111_suppl_supplementary_materials.doc]

**Supplementary Materials**

**Study Cohort**

Participants were members of the Environmental Risk (E-Risk) Longitudinal Twin Study, which tracks the development of a nationally-representative birth cohort of 2232 British twin children. The sample was drawn from a larger cohort of twins born in England and Wales in 1994-1995.^1^ Full details about the sample are reported elsewhere.^2^ Briefly, the E-Risk sample was constructed in 1999-2000, when 1116 families with same-sex 5-year-old twins (93% of those eligible) participated in home-visit assessments. Families were recruited to represent the UK population of families with newborns in the 1990s, based on residential location throughout England and Wales and mothers’ age. Teenaged mothers with twins were over-selected to replace high-risk families who were selectively lost to the register through non-response. Older mothers having twins via assisted reproduction were under-selected to avoid an excess of well-educated older mothers. E-Risk families are representative of UK households across the spectrum of neighborhood-level deprivation: 25.6% of E-Risk families live in “wealthy achiever” neighborhoods compared to 25.3% of households nation-wide; 5.3% vs 11.6% live in “urban prosperity” neighborhoods; 29.6% vs 26.9% live in “comfortably off” neighborhoods; 13.4% vs 13.9% live in “moderate means” neighborhoods; and 26.1% vs 20.7% live in “hard-pressed” neighborhoods.^3,4^ E-Risk families under-represent “urban prosperity” neighborhoods because such households are likely to be childless. The sample comprised 56% monozygotic and 44% dizygotic twin pairs, and sex was evenly distributed within zygosity (49% male). All families were English speaking, and the majority (93.7%) were White. Follow-up home visits were conducted when children were 7 years (98% of the 1116 E-Risk Study families participated), 10 years (96% participation) and 12 years (96% participation). Home visits at ages 5, 7, 10, and 12 years included assessments with participants as well as their mother (or primary caretaker). Each twin participant was assessed by a different interviewer. The Joint South London and Maudsley and the Institute of Psychiatry Research Ethics Committee approved each phase of the study. Parents gave informed consent and children gave assent.

**Measure of psychotic symptoms**

E-Risk families were visited by mental health trainees or professionals when children were aged 12.^5^ Each child was privately interviewed about 7 psychotic symptoms pertaining to delusions and hallucinations, with items including “have other people ever read your thoughts?,” “have you ever thought you were being followed or spied on?,” and “have you ever heard voices that other people cannot hear?.” This interview has been described in detail previously.^5^ The item choice was guided by the Dunedin Study’s age-11 interview protocol^6^ and an instrument prepared for the Avon Longitudinal Study of Parents and Children.^7^ Interviewers coded each experience 0, 1, 2 indicating respectively “not a symptom,” “probable symptom,” and “definite symptom.” A conservative approach was taken in designating a child’s report as a symptom. First, the interviewer probed using standard prompts designed to discriminate between experiences that were plausible (e.g., “I was followed by a man after school”) and potential symptoms (e.g., “I was followed by an angel who guards my spirit”), and wrote down the child’s narrative description of the experience. Second, items and interviewer notes were assessed by a psychiatrist expert in schizophrenia, a psychologist expert in interviewing children, and a child and adolescent psychiatrist to verify the validity of the symptoms. Third, because children were twins, experiences limited to the twin relationship (e.g., “My twin and I often know what each other are thinking”) were coded as “not a symptom”. Children were only designated as experiencing psychotic symptoms if they reported at least one definite symptom. At age 12, 5.9% (*N* = 125) of children reported experiencing psychotic symptoms. This is similar to the prevalence of psychotic symptoms in other community samples of children and adolescents.^8-12^ Furthermore, we have previously shown that childhood psychotic symptoms in this cohort have good construct validity, sharing many of the genetic, social, neurodevelopmental, and behavioral risk factors and correlates as adult schizophrenia.^5^

**Measures of victimization**

Exposure to several types of victimization was assessed repeatedly when the children were 5, 7, 10, and 12 years of age and dossiers have been compiled for each child with cumulative information about exposure to domestic violence between the mother and her partner; frequent bullying by peers; physical maltreatment by an adult; sexual abuse; emotional abuse; and neglect. The E-Risk team has previously reported evidence on the reliability and validity of the measures of domestic violence,^13^ bullying,^14,15^ physical maltreatment and sexual abuse,^16^ emotional abuse,^17^ and physical neglect.^18^ All the component measures are outlined briefly below.

*Physical Domestic Violence.* Mothers reported about perpetration of and victimization by 12 forms of physical violence (e.g., slapping, hitting, kicking, strangling) from the Conflict Tactics Scale,^19^ on three assessment occasions during the child's first decade of life (when the children were 5, 7, and 10 years of age). Reports of either perpetration or victimization constituted evidence of physical domestic violence. Families in which no physical violence took place were coded as 0 (55.2%); families in which physical violence took place on one occasion were coded as 1 (28.0%); and families in which physical violence took place on multiple occasions were coded as 2 (16.8%).

*Bullying by Peers.* Experiences of victimization by bullies were assessed using both mothers’ and children’s reports. During the interview, the following standard definition of bullying was read out: *“Someone is being bullied when another child (a) says mean and hurtful things, makes fun, or calls a person mean and hurtful names; (b) completely ignores or excludes someone from their group of friends or leaves them out on purpose; (c) hits, kicks, or shoves a person, or locks them in a room; (d) tells lies or spreads rumours about them; and (e) other hurtful things like these. We call it bullying when these things happen often, and when it is difficult to make it stop. We do not call it bullying when it is done in a friendly or playful way.”* Mothers were interviewed when children were 7, 10, and 12 years old and asked whether either twin had been bullied by another child, responding never, yes, or frequently. We combined mothers’ reports when children were age 7 and 10 to derive a measure of victimization during primary school. Mothers’ reports when the children were 12 years old indexed victimization during secondary school. During private interviews with the children when they were 12 years old, the children indicated whether they had been bullied by another child during primary or secondary school. When a mother or a child reported victimization, the interviewer asked them to describe what happened. Notes taken by the interviewers were later checked by an independent rater to verify that the events reported could be classified as instances of bullying operationally defined as evidence of (a) repeated harmful actions, (b) between children, and (c) where there is a power differential between the bully and the victim.^15^ Although inter-rater reliability between mothers and children was only modest (*kappa* = 0.20-0.29), reports of victimization from both informants were similarly associated with children’s emotional and behavioural problems, suggesting that each informant provides a unique but meaningful perspective on bullying involvement.^15^ We thus combined mother and child reports of victimization to capture all instances of bullying victimization for primary and secondary school separately: reported as not victimized by both mother and child; reported by either mother or child as being occasionally victimized; and reported as being occasionally victimized by both informants or as frequently victimized by either mother or child or both.^20^ We then combined these primary and secondary school ratings to create a bullying victimization variable for the entire childhood period (5-12 years). Children who were never bullied in primary or secondary school or occasionally bullied during one of these time periods were coded as 0 (55.5%); children who were occasionally bullied during primary and secondary school, or frequently bullied during one of these time periods were coded as 1 (35.6%); and children who were frequently bullied at both primary and secondary school were coded as 2 (8.9%).

*Physical and sexual harm by an adult.* We assessed childhood physical and sexual harm in the E-Risk Study using an approach that resembles the process undertaken by child protection agencies. Essentially this is a two-stage process. In child protection, professionals such as teachers working with children typically raise concerns if they observe signs or symptoms or if they become aware of risk that children are victims of violence. When concerns are raised, child protection officers then review the concerns and evaluate them in the context of information previously gathered on that child or family in order to determine the likelihood that abuse has taken place. In the E-Risk Study, research workers visited the home in pairs, and were extensively trained to detect signs of abuse or neglect. Each time the two research workers visited a home, they interviewed the mother using a structured interview about child harm, tested the children, and observed the family environment using the Home Observation for Measurement of the Environment (HOME).^21^ If either research worker had any concerns, they flagged up the case for review. Immediately after each home visit, a review was performed if a family was flagged. In addition, at each wave, any family who had been flagged on a prior wave of the study was automatically reviewed again. The reviews were performed independently by at least 2 clinical psychologists or psychiatrists, and were based on comprehensive dossiers compiled across multiple home visits for each study member during the course of the ongoing longitudinal study.

At age 5, assessments were based on the standardised clinical protocol from the MultiSite Child Development Project.^22,23^ At ages 7, 10, and 12 this interview was modified to expand its coverage of contexts for child harm. Interviews were designed to enhance mothers’ comfort with reporting valid child maltreatment information, while also meeting researchers’ responsibilities for referral under the UK Children Act. Specifically, mothers were asked whether either of their twins had been intentionally harmed (physically or sexually) by an adult or had contact with welfare agencies. If caregivers endorsed a question, research workers made extensive notes on what had happened, and indicated whether physical and/or psychological harm had occurred. Under the U.K. Children Act, our responsibility was to secure intervention if maltreatment was current and ongoing. Such intervention on behalf of E-Risk families was carried out with parental cooperation in all but one case. No families left the study following intervention.

Over the years of data collection, the study developed a cumulative profile for each child, comprising the caregiver reports, recorded debriefings with research workers who had coded any indication of maltreatment at any of the successive home visits, recorded narratives of the successive caregiver interviews, and information from clinicians whenever the Study team made a child-protection referral. Each time we visited a home, the research workers flagged concerns, and if there was sufficient evidence to code definite harm then, we did so. If evidence only met the level of probable harm, we kept an “ongoing concern list” and if, at a later wave, there was continued evidence of probable harm, or new evidence, the code was upgraded to definite harm. The profiles were reviewed at the end of the age-12 phase by at least two clinical psychologists or psychiatrists. Initial inter-rater agreement between the coders was 90% in cases for whom maltreatment was identified (100% for cases of sexual abuse), and discrepantly coded cases were resolved by consensus review. These were coded as: 0 = no physical harm at any age; 1 = probable physical harm at any age; and 2 = definite physical harm at any age. There were 15.0% of children coded as probably being exposed to physical harm and 5.1% as definitely physically harmed by 12 years of age. There were 1.5% of the children coded as being exposed to sexual abuse.

*Emotional abuse and neglect.* These forms of maltreatment were coded from research workers’ narratives of the home visits at ages 5, 7, 10, and 12. We coded quite severe examples of parental behavior observed. For example, a mother who had schizophrenia screamed and swore at the children throughout the home visit. As another example, a father who was drunk during the home visit repeatedly spoke abusively to the children in front of the research workers. We found that coders could not empirically separate emotional abuse and emotional neglect in a reliable way and thus such experiences were coded together as emotional abuse/neglect. Inter-rater agreement between the coders exceeded 85% for cases with emotional abuse and neglect, and discrepant cases were resolved by consensus review. Children with no evidence of emotional abuse/neglect were coded as 0 (88.3%), those where there was some indication of emotionally inappropriate/potentially abusive or neglectful behavior were coded as 1 (8.7%), and where there was evidence of severe emotional abuse/neglect the children were coded as 2 (3.0%).

*Physical neglect.* The cumulative observations of the physical state of the home environment documented by the research workers during home visits to the twins at ages 5, 7, 10 and 12 were reviewed by two raters for evidence of physical neglect. This was defined as any sign that the caretaker was not providing a safe, sanitary, or healthy environment for the child. This included the child not having proper clothing or food, as well as grossly unsanitary home environments. (However, this did not include a family living in a crime-ridden neighborhood for economic reasons.) Inter-rater agreement between the coders exceeded 85%, and discrepantly coded cases were resolved by consensus review. Children with no evidence of physical neglect were coded as 0 (90.9%), those for whom there was an indication of minor physical neglect were coded as 1 (7.1%), and where there was evidence of severe physical neglect the children were coded as 2 (2.0%).

*Childhood poly-victimization.* Finkelhor et al^24^ operationalize poly-victimisation as the total number of victimization types that a child experiences. The E-Risk poly-victimization variable was derived by summing all victimization experiences that received a code of ‘2’: 73.5% of children had zero victimization experiences; 20.1% had 1 victimization experience; 3.8% had 2 victimization experiences; 1.8% had 3 victimization experiences; 0.8% had 4 victimization experiences; and 0.1% had 5 victimization experiences. For the current analysis, we dichotomized the poly-victimization variable into those children having zero or one victimization experience (N = 1987, 93.4%), and those who had 2 or more victimization experiences (N = 140, 6.6%), who had completed the psychotic symptoms interview at age 12.

**References**

1. Trouton A, Spinath FM, Plomin R. Twins Early Development Study (TEDS): a multivariate, longitudinal genetic investigation of language, cognition and behavior problems in childhood. *Twin Res*. 2002;5(5):444-448.

2. Moffitt TE, E-Risk Study Team. Teen-aged mothers in contemporary Britain. *J Child Psychol Psychiatry*. 2002;43(5):1-16.

3. CACI. *ACORN user guide*. London, UK: CACI Information Services; 2006.

4. Caspi A, Taylor A, Moffitt TE, Plomin R. Neighborhood deprivation affects children’s mental health: environmental risks identified in a genetic design. *Psychol Sci*. 2000;11(4):338-342.

5. Polanczyk G, Moffitt T, Arseneault L, et al. Etiological and clinical features of childhood psychotic symptoms. *Arch Gen Psychiatry.* 2010;67(4):328-338.

6. Poulton R, Caspi A, Moffitt TE, et al. Children’s self-reported psychotic symptoms and adult schizophreniform disorder: a 15-year longitudinal study. *Arch Gen Psychiatry.* 2000;57:1053-1058.

7. Schreier A, Wolke D, Thomas K, et al. Prospective study of peer victimization in childhood and psychotic symptoms in a non-clinical population at age 12 years. *Arch Gen Psychiatry.* 2009;66:527-536.

8. Kelleher I, Connor D, Clarke MC, Devlin N, Harley M, Cannon M. Prevalence of psychotic symptoms in childhood and adolescence: a systematic review and meta-analysis of population-based studies. *Psychol Med.* 2012;42:1857-1863.

9. Horwood J, Salvi G, Thomas K, et al. IQ and non-clinical psychotic symptoms in 12-year-olds: results from the ALSPAC birth cohort. *Br J Psychiatry.* 2008;193(3):185-191.

10. Yoshizumi T, Murase S, Honjo S, Kaneko H, Murakami T. Hallucinatory experiences in a community sample of Japanese children. *J Am Acad Child Adolesc Psychiatry.* 2004;43(8):1030-1036.

11. Scott J, Chant D, Andrews G, McGrath J. Psychotic-like experiences in the general community: the correlates of CIDI psychosis screen items in an Australian sample. *Psychol Med.* 2006;36(2):231-238.

12. Dhossche D, Ferdinand R, Van der Ende J, Hofstra MB, Verhulst F. Diagnostic outcome of self-reported hallucinations in a community sample of adolescents. *Psychol Med.* 2002;32:619-627.

13. Moffitt TE, Caspi A, Krueger RF, et al. Do partners agree about abuse in their relationship? A psychometric evaluation of inter-partner agreement. *Psychol Assess.* 1997;9:47-56.

14. Arseneault L, Walsh E, Trzesniewski K, et al. Bullying victimization uniquely contributes to adjustment problems in young children: a nationally representative cohort study. *Pediatr.*  2006;118:130-138.

15. Shakoor S, Jaffee SR, Andreou P, et al. Mothers and children as informants of bullying victimization: results from an epidemiological cohort of children. *J Abnorm Child Psychol.* 2011;39:379-387.

16. Jaffee SR, Caspi A, Moffitt TE, et al. Physical maltreatment victim to antisocial child: evidence of an environmentally mediated process. *J Abnorm Psychol.* 2004;113:44-55.

17. Danese A, Moffitt TE, Arseneault L, et al. The origins of cognitive deficits in victimized children: implications for neuroscientists and clinicians. *Am J Psychiatry.* 2016;doi:10.1176/appi.ajp.2016.16030333.

18. Fisher HL, Caspi A, Moffitt TE, et al. Measuring adolescents' exposure to victimization: The Environmental Risk (E-Risk) Longitudinal Twin Study. *Dev Psychopathol.* 2015;27:1399-1416.

19. Straus M. Measuring intrafamily conflict and violence: the Conflict Tactics (CT) scales. In: M. A. Straus & R. G. Gelles (Eds.), *Physical violence in American families: risk factors and adaptations to violence in 8,145 families* (pp.403-424). New Brunswick, NJ: Transaction Press; 1990.

20. Bowes L, Maughan B, Ball H, et al. Chronic bullying victimization across school transitions: the role of genetic and environmental influences. *Dev Psychopathol.* 2013;25(2):333-346.

21. Bradley R, Caldwell B. Home observation for measurement of the environment: a validation study of screening efficiency. *Am J Ment Defic*. 1977;81:417-420.

22. Dodge KA, Bates JE, Pettit GS. Mechanisms in the cycle of violence. *Science.* 1990;250:1678-1683.

23. Lansford JE, Dodge KA, Pettit GS, Batesm JE, Crozier J, Kaplow J. Long-term effects of early child physical maltreatment on psychological, behavioral, and academic problems in adolescence: a 12-year prospective study. *Arch Pediatr Adolesc Med.* 2002;156:824-830.

24. Finkelhor D, Ormrod RK, Turner HA. Poly-victimization: a neglected component in child victimization. *Child Abuse Negl.* 2007;31:7-26.
